# Synthesis, structure and toxicity evaluation of ethanolamine nitro/chloronitrobenzoates: a combined experimental and theoretical study

**DOI:** 10.1186/s13065-017-0346-5

**Published:** 2017-12-06

**Authors:** Manuela Crisan, Liliana Halip, Paulina Bourosh, Sergiu Adrian Chicu, Yurii Chumakov

**Affiliations:** 1 Institute of Chemistry, Timisoara of Romanian Academy, 24 Mihai Viteazul Avenue, 300223 Timisoara, Romania; 2 Institute of Applied Physics, Academy of Sciences of Moldova, Academiei Street 5, 2028 Chisinau, Republic of Moldova; 3 Siegstr. 4, 50859 Cologne, Germany

**Keywords:** Nitrobenzoic and chloronitrobenzoic acids and derivatives, Toxicity, Single crystal X-ray diffraction, Chemical reactivity

## Abstract

**Background:**

Nitroaromatic and chloronitroaromatic compounds have been a subject of great interest in industry and recently in medical-pharmaceutic field. 2-Chloro-4-nitro/2-chloro-5-nitrobenzoic acids and 4-nitrobenzoic acid are promising new agents for the treatment of main infectious killing diseases in the world: immunodeficiency diseases and tuberculosis.

**Results:**

New ethanolamine nitro/chloronitrobenzoates were synthesized and characterized by X-ray crystallography, UV–vis, FT-IR and elementary analysis techniques. The toxicity of the compounds prepared and correspondent components was evaluated using *Hydractinia echinata* as test system. A significant lower toxicity was observed for nitro-derivative compared with chloronitro-derivatives and individual components. Crystallographic studies, together with the chemical reactivity and stability profiles resulted from density functional theory and ab initio molecular orbital calculations, explain the particular behavior of ethanolamine 4-nitrobenzoate in biological test.

**Conclusions:**

The experimental and theoretical data reveal the potential of these compounds to contribute to the design of new active pharmaceutical ingredients with lower toxicity.

**Electronic supplementary material:**

The online version of this article (10.1186/s13065-017-0346-5) contains supplementary material, which is available to authorized users.

## Introduction

Nitroaromatic and chloronitroaromatic compounds are versatile precursors, the vast majority synthetic and frequently employed as important intermediates for the synthesis of industrial chemicals, dyes, pigments and pharmaceutical drugs [1–3]. The functional groups nitro and chloride provide chemical and structural diversity and a significant impact on properties and reactivities of chemicals, making these compounds attractive in different research fields over the past decades. Many pharmaceuticals have their chemical origins in nitro- and chloronitroaromatic compounds. They are used to treat a wide variety of diseases categories: Parkinson, angina, insomnia and parasitic infection (e.g. *Giardiasis, Amebiasis, Trichomoniasis*) [4–6]. Recently, 4-nitrobenzoic acid (4-NO_2_BA), 2-chloro-4-nitrobenzoic acid (2-Cl-4-NO_2_BA) and 2-chloro-5-nitrobenzoic acid (2-Cl-5-NO_2_BA) have been used as active ingredients in the main infectious killing diseases in the world. Therefore, 4-NO_2_BA is used as inhibitor agent for identification of *Mycobacterium tuberculosis* complex [7–9] and 2-Cl-4-NO_2_BA/2-Cl-5-NO_2_BA used in a novel therapy for immunodeficiency diseases, including the human immunodeficiency virus (HIV) infection [10, 11].

Taking into account the prospects of nitro- and chloronitrobenzoic acids and the fact that about a half of all active pharmaceutical ingredients are used today as salts due to their improved drug’s physicochemical properties, we have focused to prepare new nitro/chloronitroderivatives with lower toxicity. Limited information on the experimental toxicity profile of benzoic agents have been founded in literature [12–15]. Experimental and/or theoretical toxicity studies on different organisms are required for new derivatives of an active pharmacological substance. The safety profile of a salt depends significantly on the cation nature and the alkyl side chain. The majority of hydrogen-bonded complexes of nitro-, respectively chloronitrobenzoic acids use heterocyclic amines (e.g. pyridine, piperazine, morpholine), considered strong mutagens [16–18].

This study proposes the development of new compounds with dual biological activity, based on biologically active components: ethanolammonium as cation and 2-chloro-4-nitrobenzoate, 2-chloro-5-nitrobenzoate and respectively 4-nitrobenzoate as anions. Ethanolamine (EA) is a naturally occurring component and a suitable model of alkanolamines, which is safer, economical and commercially available, used as base chemical in the production of pharmaceuticals. It is an essential component of cell membranes, present in the synthesis of membrane lipids, such as phosphatidylethanolamine and phosphatidylcholine [19]. Recent studies introduce EA as a new therapeutic agent in the treatment of age-associated human diseases, being involved in autophagy regulation [20].

Besides the plethora of properties noted above, both EA and substituted benzoic acids have the ability to establish strong and directional hydrogen bonds, forming supramolecular systems with different topologies, important in crystal engineering [21–23]. Continuing our interest in multicomponent organic crystal with dual biological activity, some of the major objectives of this paper are to obtain crystalline ethanolamine salts of 2-Cl-4-NO_2_BA (1), 2-Cl-5-NO_2_BA (2) and 4-NO_2_BA (3), to characterize them physico-chemically and structurally, and to investigate their supramolecular synthons and molecular packing. A comparative analysis on toxicity of the compounds studied is also presented, using *Hydractinia echinata*, previously demonstrated to be an excellent test system [12, 24]. In order to explain the particular behavior of ethanolamine salts of nitro- and chloronitrobenzoic acids in biological test, a theoretical study regarding the chemical reactivity and stability profiles was described in correlation with their biological activity (toxicity) and X-ray structures.

## Experimental

### General

All the chemicals used for the synthesis were of analytical grade and purchased from Fluka AG (Buchs SG). EA was freshly distilled before any use. Melting points were determined on Boetius melting point apparatus and are uncorrected. FT-IR spectra of new synthesized compounds were recorded as KBr pellet on a JASCO—FT/IR-4200 spectrometer, in the range 4000–400 cm^−1^, with a resolution of 4.0 cm^−1^ and a scanning speed of 16 mm s^−1^. The optical properties were examined by using a UV–vis spectrophotometer at room temperature. UV–vis spectra in the 190–800 nm range were measured on PERKIN-ELMER LAMBDA 12 UV–vis spectrometer.

### Synthesis and characterization of compounds 1–3

Title compounds were prepared in a 1:1 molar ratio via proton exchange reaction by mixing the diethyl ether solutions of ethanolamine and 2-Cl-4-NO_2_BA/2-Cl-5-NO_2_BA, respectively 4-NO_2_BA. The mixture was stirred and heated to reflux for 30 min to complete the reaction. The yellowish solutions obtained were kept for slow evaporation. After a few days, crystals suitable for X-ray diffraction were obtained. The crystals were filtered and washed with diethyl ether and dried in air. The reaction yields were about 90–92%. The purity of the obtained compounds ranged between 99.1 and 99.4%, established by an UV spectrophotometric method [25] and confirmed by elemental analysis. The FT-IR spectra for each compound were consistent with salt formation.

1. C_9_H_12_ClN_2_O_5_ (262.58), m.p. 112–115 °C; λ_max_ = 277.93 nm; FT-IR spectra (KBr pelet, cm^−1^): 3194 (νNH_3_^+^), 1613 (ν_as_COO^−^), 1520 (δNH_3_^+^), 1394 (ν_s_COO^−^), 1065 (νC–O), calcd. (%): C 41.13, H 4.57, Cl 13.52, N 10.66; found (%): C 41.04, H 4.41, Cl 13.39, N 10.51;
2. C_9_H_12_ClN_2_O_5_·H_2_O (280.58), m.p. 116–118 °C; λ_max_ = 279.81 nm; FT-IR spectra (KBr pelet, cm^−1^): 3166 (νNH_3_^+^), 1621 (ν_as_COO^−^), 1519 (δNH_3_^+^), 1404 (ν_s_COO^−^), 1087 (νC–O), calcd. (%): C 38.49, H 4.99, Cl 12.65, N 9.98; found (%): C 38.39, H 4.82, Cl 12.48, N 9.84;
3. C_9_H_12_N_2_O_5_ (228.20), m.p. 173–175 °C; λ_max_ = 273.55 nm; FT-IR spectra (KBr pelet, cm^−1^): 2955 (νNH_3_^+^), 1648 (ν_as_COO^−^), 1515 (δNH_3_^+^), 1392 (ν_s_COO^−^), 1018 (νC–O), calcd. (%): C 47.33, H 5.25, N 12.27; found (%): C 47.18, H 5.13, N 12.09;

### Single crystal X-ray structure analysis

**Fig. 1:**
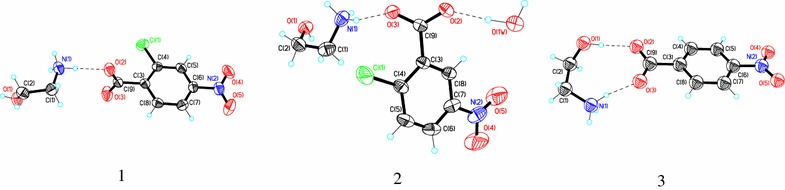
An ORTEP view of the compounds 1–3 showing the atom-numbering scheme. Displacement ellipsoids are drawn at the 50% probability level and the charge-assisted hydrogen bonds are represented by dashed lines

The X-ray data sets were collected at room temperature on a Siemens P3/PC diffractometer equipped with CuKα-radiation. The unit cell parameters were determined, and the structures refinement were performed using the SHELX-97 program [26]. Non hydrogen atoms have been refined with anisotropic displacement. Hydrogen atoms were found from differential Fourier maps and refined without any constrains. Hydrogen atoms that are not involved in hydrogen bonding were omitted from the representation of crystal packings. The crystals remained stable throughout the data collection. Drawings of the structures of 1–3 compounds were produced using ORTEP program (Fig. 1) [27].

Crystallographic data and refinement for compounds 1–3 were summarized in Table 1.

**Table 1:** Crystal data and structure refinement parameters for compounds 1–3

Compound	1	2	3^a^
Empirical formula	C_9_H_11_ClN_2_O_5_	C_9_H_13_ClN_2_O_6_	C_9_H_12_N_2_O_5_
Formula weight	262.65	280.66	228.21
Temperature (K)	293 (2)	293 (2)	293 (2)
Wavelength (Å)	1.54184	1.54184	0.71073
Crystal system	monoclinic	triclinic	monoclinic
Space group	*P*2_1_ */c*	*P*-1	*P*2_1_ */c*
Z	4	2	4
Unit cell dimensions			
*a* (Å)	8.402 (2)	7.019 (1)	6.211 (2)
*b* (Å)	6.334 (1)	9.512 (2)	8.651 (3)
*c* (Å)	22.023 (4)	9.740 (2)	19.594 (5)
α (°)	90	79.19 (3)	90
β (°)	100.72 (3)	88.58 (3)	91.27 (2)
γ (°)	90	78.42 (3)	90
*V* (Å^3^)	1151.6 (4)	625.7 (2)	1052.6 (5)
*D*_calc_ (g cm^−3^)	1.515	1.490	1.440
μ (mm^−1^)	3.099	2.950	0.119
*F* (000)	544	292	480
Crystal size (mm)	0.22 × 0.14 × 0.07	0.15 × 0.12 × 0.08	0.35 × 0.4 × 0.6
θ range for data collection (°)	4.09–70.07	4.62–70.01	2.08–27.06
Limiting indices	− 10 ≤ *h* ≤ 1	− 8 ≤ *h* ≤ 8	0 ≤ *h* ≤ 7
	− 7 ≤ *k* ≤ 0	− 4 ≤ *k* ≤ 11	0 ≤ *k* ≤ 11
	− 26 ≤ *l* ≤ 26	− 11 ≤ *l* ≤ 11	− 25 ≤ *l* ≤ 25
Reflections collected/unique	2336/2192	2370/2370	2501/2294
Reflections with [*I* > 2σ(*I*)]	2010	2282	1761
Parameters	156	165	193
Goodness-of-fit on F^2^	1.007	1.003	1.001
*R*_1_, w*R*_2_ [*I* > 2σ(*I*)]	0.0384, 0.1128	0.0415, 0.1175	0.0371, 0.1023
*R*_1_, w*R*_2_ (all data)	0.0414, 0.1163	0.0424, 0.1187	0.0540, 0.11068
Largest difference in peak and hole (e Å^−3^)	0.330/− 0.311	0.385/− 0.315	0.265/− 0.212

^a^Crystallographic data of compound 3 [21] is presented here for comparison with compounds 1 and 2

Crystallographic data were deposited in the Cambridge Crystallographic Data Centre (CCDC Numbers 853484, 853485, 257807).

### Toxicity test

The toxicity of compounds 1–3 and the correspondent components was evaluated using *H. echinata* as test system. This simple and common invertebrate living in European and North American coastal areas is rapidly reproductible and considered sustainable for in vivo experiments. The used method was identical with the one described in previous articles [12, 24]. Dishes with 30 *H. echinata* larvae were exposed to 3 mL seawater (980 mosmol, pH 8.2, 18 °C) containing 20 mM CsCl and the test compounds were added for 3 h. The concentration (mol L^−1^) at which the frequency of metamorphosis induction larvae to polyp was reduced by 50% with respect to control was determined after 24 h and noted as MRC_50_ (metamorphosis reducing concentration) and correspond to EC_50_ (50-effective concentration) in literature. The measured value of toxicity (M) was showed as logarithm of reciprocal value of MRC_50_ (M = log 1/MRC_50_). Triplicate experiments were performed for each concentration assessment of title compounds and each experiment was repeated twice.

### Computational methods

All theoretical calculations were performed using the Jaguar 8.9 quantum program suite [28–31]. Single point and lowest-energy calculations were performed using Hartree–Fock theory and 6.31G** basic set. The crystal structures were visualized and prepared for calculations using Maestro10.3 (Schrödinger) with MacroModel10.9 (Schrödinger). According to Koopmans’s theorem [32] the energies of highest occupied (HOMO) and lowest (LUMO) unoccupied molecular orbitals were used to determine the ionization potential (I), and electron affinity (A) as in Eqs. (1) and (2). Accordingly, their derived parameters as band gap, electrophilicity (ω), hardness (η), chemical potential (µ), electronegativity (χ) and electrofilicity (ω) were calculated using Eqs. 3–6.

1 $$ I \approx - \varepsilon_{HOMO} $$

2 $$ A \approx - \varepsilon_{LUMO} $$

3 $$ \eta \approx \frac{I - A}{2} \approx \frac{{\varepsilon_{LUMO} - \varepsilon_{HOMO} }}{2} $$

4 $$ \mu \approx - \frac{I + A}{2} \approx \frac{{\varepsilon_{LUMO} + \varepsilon_{HOMO} }}{2} $$

5 $$ \chi \approx \frac{I + A}{2} \approx \frac{{ - \varepsilon_{HOMO} - \varepsilon_{LUMO} }}{2} $$

6 $$ \omega = \frac{{\mu^{2} }}{2\eta } $$

## Results and discussion

### Synthesis and characterization

The title compounds 1–3 are stable in air at room temperature, and have been formed in accordance with the appropriate “rule of three” that serves as a guide for salt formation by determining the extent of proton transfer (Table 2). The principle of this rule is based on values of ΔpKa = pKa (protonated base) − pKa (acid) which is a tool for predicting salt or co-crystal formation. For values of ΔpKa greater than 3 a molecular salt is formed, while for values less than 0 a co-crystal is formed [33]. For an intermediate value, no precise prediction can be made [34]. The melting points of compounds 1–3 are well defined, being lower than the corresponding acids (2-Cl-4-NO_2_BA—m.p. 142 °C, 2-Cl-5-NO_2_BA—m.p. 166 °C, 4-NO_2_BA—m.p. 237 °C). The UV–vis spectral measurements indicate the cut off wavelength of 277.93, 279.81 nm, respectively 273.55 nm, and no characteristic absorptions in visible region were observed (Additional file 1: Figure S1).

**Table 2:** Tabulated ΔpKa and hydrogen-bonding geometry (Å, °) for compounds 1–3

ΔpKa	D–H^…^A	d(D–H)	d(H^…^A)	d(D^…^A)	(DHA)	Symmetry transformation for H-acceptor
**1**						
7.58	N(1)–H(2)^…^O(2)	0.89	1.86	2.747 (2)	173	*x*, *y*, *z*
	N(1)–H(1)^…^O(2)	0.89	2.12	2.815 (2)	135	*x* + 1, *y* + 1, *z* + 1
	N(1)–H(3)^…^O(1)	0.89	2.20	2.868 (2)	131	− *x*, − *y* + 1, − *z* + 1
	O(1)–H(1)^…^O(3)	0.82	1.87	2.681 (2)	169	− *x*, − *y* + 1, − *z* + 1
**2**						
7.33	N(1)–H(1)^…^O(3)	0.89	1.90	2.766 (2)	164	*x*, *y*, *z*
	N(1)–H(2)^…^O(1)	0.89	1.96	2.798 (2)	156	− *x*, − *y*, − *z*
	N(1)–H(3)^…^O(1W)	0.89	1.98	2.858 (2)	171	− *x* + 1, − *y*, − *z* + 1
	O(1)–H(1)^…^O(3)	0.82	1.90	2.716 (2)	172	*x* − 1, *y*, *z*
	O(1W)–H(1)^…^O(2)	0.97	1.99	2.857 (2)	149	− *x* + 2, − *y*, − *z* + 1
	O(1W)–H(2)^…^O(2)	1.05	1.71	2.754 (2)	175	*x*, *y*, *z*
**3** ^a^						
6.07	N(2)–H(2)^…^O(1)	0.96	1.81	2.738	168	*x*, *y*, *z*
	O(5)–H(5)^…^O(2)	0.87	1.81	2.683	177	*x*, *y*, *z*
	N(2)–H(2)^…^O(5)	0.94	1.88	2.788	162	− *x* − 1, + *y* − 1/2, − *z* + 1/2
	N(2)–H(2)^…^O(2)	0.94	1.81	2.757	170	*x* + *1*, *y*, *z*

D and A are hydrogen bond donor and acceptor atoms. ΔpKa = pKa (base) − pKa (acid) were calculated using the pKa data from Ref. [38]. All pKa values have been determined in aqueous solutions

^a^Data of compound 3 [21] are presented here for comparison with compounds 1 and 2

The IR spectra provide the evidence of salt formation by the presence of absorption bands in the regions 1650–1540 and 1450–1360 cm^−1^, arising from the asymmetric and symmetric vibrations of the COO^−^ group and by the absence of bands at 1710–1680 cm^−1^, corresponding to carbonyl stretch (ν_C=O_) and 1320–1210 cm^−1^ characteristic for ν_C–OH_ vibrations in a COOH group (Additional file 1: Figure S2) [35, 36]. The appearance of C–O vibrations at 1100–1000 cm^−1^, belonging to –CH_2_OH from EA was a supplementary proof of salt formation. The asymmetric –NH_3_^+^ stretching vibrations are observed in the region 3200–2800 cm^−1^ and weak bands of symmetric stretching –NH_3_^+^ near 2600 and 2100 cm^−1^. A strong –NH_3_^+^ deformation band is observed at 1550–1485 cm^−1^, which almost overlaps with asymmetric vibration of –NO_2_ group at 1570–1485 cm^−1^.

### Crystal structure

**Fig. 2:**
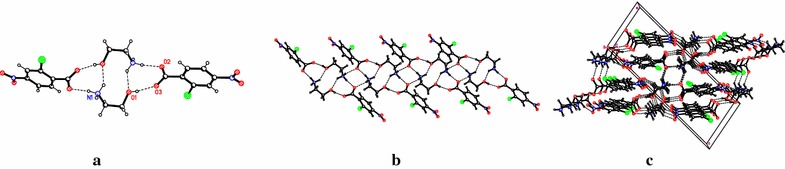
Intramolecular hydrogen bonds resulting in synthons, layer and crystal packing in compound 1. **a** The R^4^_4_(18) synthon formed by hydrogen bonds in compound 1; **b** the chains formed by hydrogen bonds in 1; **c** the crystal packing of 1 showing the weak interactions between the chains via C–O hydrogen bonds

**Fig. 3:**
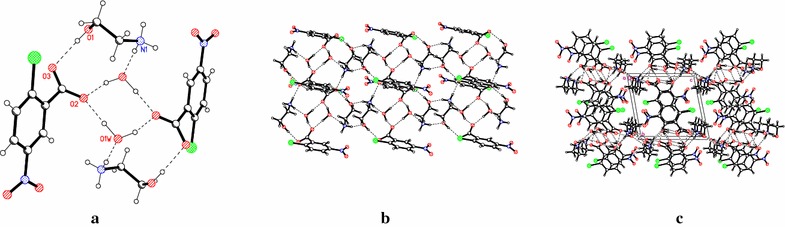
Intramolecular hydrogen bonds resulting in synthons, layer and crystal packing in compound 2. **a** The R^3^_3_(11) synthons linked by synthon R^4^_2_(8) formed by hydrogen bonds in compound 2; **b** the layer formed by hydrogen bonds in 2; **c** the crystal packing of 2 showing the arrangement of the layers in the crystal

The structural aspects of compounds 1 and 2 (Additional files 2 and 3) were investigated and compared with those of compound 3 [21]. X-ray study confirms that the proton transfer has occurred in both components, from the carboxyl group of acid to the amino group of ethanolamine, and the crystals are formed by two ionic species, anion and cation. In chloronitro-compounds (1 and 2), the basic components are connected only by one hydrogen bond (HB) (Figs. 1, 2a, b, 3a, b and Table 2), while in nitro-compound (3) by two charged-assisted HB (Fig. 1). The ionic N–H^…^O hydrogen bond plays the key role in formation of these pairs, being present in all investigated compounds. The geometric parameters for hydrogen bonds are given in Table 2. Bond lengths (Ǻ) and angles (°) for compounds 1–3 are listed in Additional file 1: Table S1.

The anions in all compounds studied form a non-planar systems, more evident in compounds 1 and 2, where the dihedral angles between the least-square planes of the phenyl rings C(3)C(4)C(5)C(6)C(7)C(8) and the least-square planes of the –COO^−^ and –NO_2_ groups are equal to 82.5, 11.0° and 47.6, 10.4°, while for compound 3 these values are 5.8 and 4.2° respectively. The cation adopts the—Syn-Clinal conformation, the N(1)C(1)C(2)O(1) torsion angles in compounds 1 and 2 being equal to − 63.2° and 51.5°, respectively, towards compound 3 where is 77.4°.

In compound 1, the system of hydrogen bridges is formed only by the –NH_3_^+^ and –OH groups from ethanolamine and carboxylate oxygen atoms. In this crystal, two cations and two anions are held together by two N–H^…^O hydrogen bonds (N(1)^…^O(2) 2.747(2) Å) and two O–H^…^O bonds (O(1)^…^O(3) 2.682(2) Å) (Fig. 2a). The *R* ^4^_4_(18) synthon is stabilized by two intermolecular hydrogen bonds N–H^…^O (N(1)^…^O(1) 2.868(2) Å). These synthons form infinite chains by four hydrogen bonds N–H^…^O (N(1)^…^O(2) 2.815(2) Å) (Fig. 2b), which are further consolidated into *2*-*D* layers through C–H^…^O hydrogen bonds (where O is one oxygen atom from –NO_2_ group of benzoate) developed along *y* direction (Fig. 2c). It is notable that a chlorine atom and the other oxygen atom from the NO_2_-group of benzoate are not involved in the crystalline structure.

Compound 2 differs from compound 1 by the position of the substituent atoms, and this compound crystallizes as a hydrate, which changes the system of hydrogen bonds (Table 2). Thus, in addition to the system of hydrogen bonds formed by the –NH_3_^+^ and –OH groups from ethanolamine and carboxylate oxygen atoms, a water molecule is involved (Fig. 1). Water molecule acts as donor in the hydrogen bond with anion and as acceptor in hydrogen bond with cation. So, the nets contain *R* ^3^_3_(11) synthons formed by three hydrogen bonds (N(1)^…^O(1W) 2.858(2) Å), O(1)^…^O(3) 2.716(2) Å and O(1W)^…^O(2) 2.754(2) Å) linked between them by the R^4^_2_(8) synthons formed by two O–H^…^O bonds (O(1W)^…^O(2) 2.754(2) Å and O(1W)^…^O(2) 2.857(2) Å). The cations, anions, and water molecules form hydrogen bonded chains, which are further hydrogen bonded to one another by pairs of water molecules, to form layers (Fig. 3a, b). The layers formed in this way stack along the *y* axis (Fig. 3c).

In the crystal structure of compound 3, the anion and cation are held together by two charge-assisted hydrogen bonds and form the infinite helix-like chains along *b* direction [21]. These chains are joined by glide plane in double chains through the C(1)–H^…^O(5) H-bonds (check labeling). Thus in the crystal packing of compounds 1 and 2, the anions and cations are self-assembled via N–H^…^O, O–H^…^O hydrogen bonds to form the chains. These ones are consolidated into 2-*D* layers by C–H^…^O H-bonds and water molecules respectively, while the crystal packing of three adopts the chain-like structure. The anions in compounds 1 and 2 form essentially non-planar systems in comparison with that in compound 3.

### Experimental determination and theoretical investigation of toxicity behaviour

The influence of the compounds studied against the transformation from larva to polyp of marine organism *H. echinata* was evaluated. The measured values of toxicity were found to be 3.22 logarithm unites (log u.), respectively 3.10 log u. in the case of chloronitro-compounds (1, 2) and 1.78 log u. for nitro-compound (3). This situation is of particular interest, because compound 3 shows a much lower toxicity (1.78 log u.) even if compared to individual components (4-NO_2_BA: 3.11 log u. and EA: 2.67 log u.).

With this purpose we proceed to investigate the molecular structures of the three compounds using ab initio methods given that the quantum chemical descriptors have shown good correlations with biological activity since the later is dependent on the nature of the compound. Using the frontier orbitals values, the energy gap and several global reactivity descriptors, such as chemical hardness (η), chemical potential (µ), electronegativity (χ) and electrophilicity index (ω), were calculated to evaluate the chemical reactivity and the stability of these compounds in order to elucidate a possible mechanism of action for this type of compounds.

**Fig. 4:**
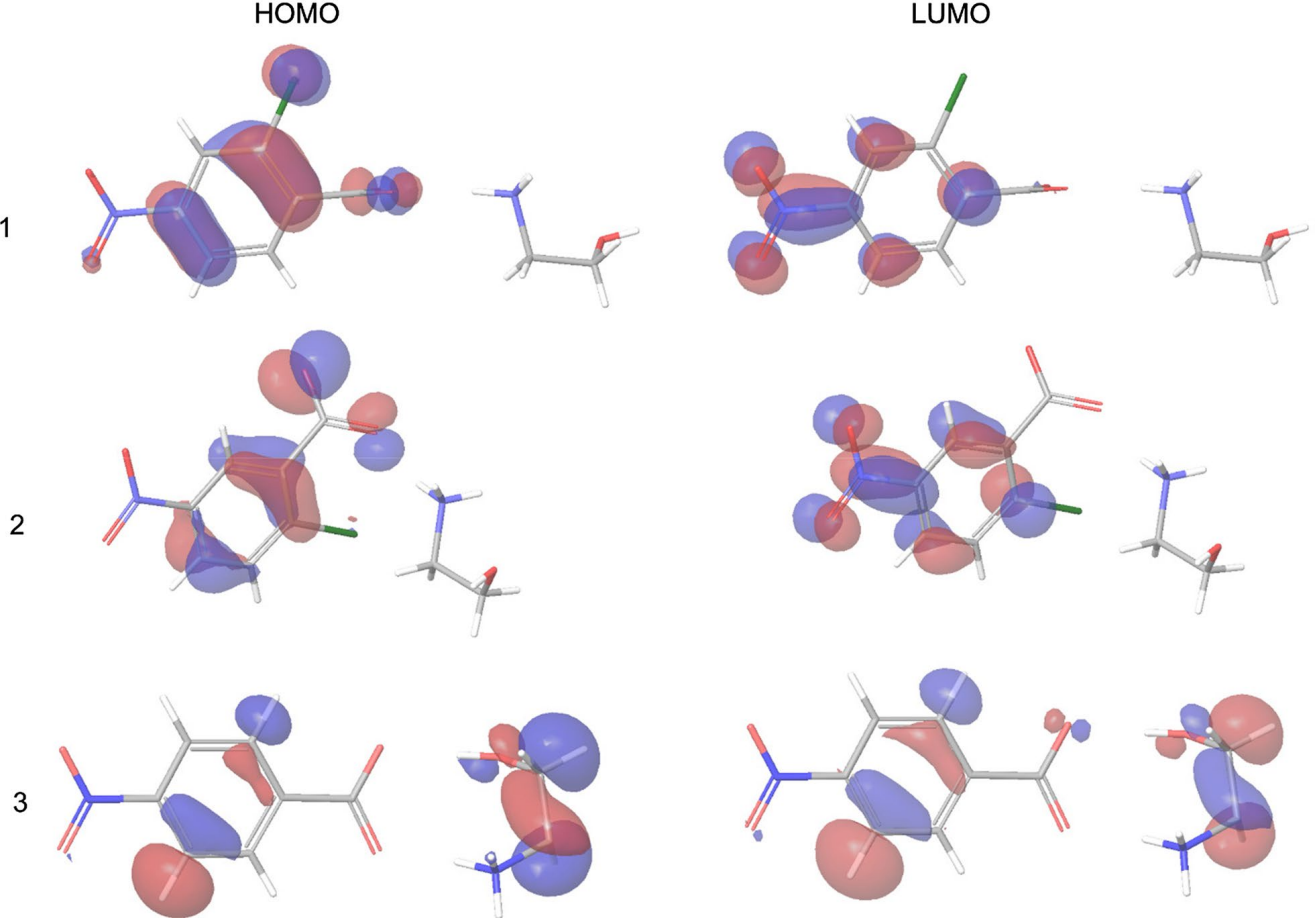
HOMO and LUMO orbitals for compounds 1–3

The locations and values of HOMO and LUMO orbitals (Fig. 4) offer important evidences about the capability of a molecule to donate or to attract electrons. Compounds 1 and 2 have both first HOMO and LUMO orbitals distributed only on the anion. Compound 3 has a higher HOMO energy value which corresponds to a stronger electron-donating capacity, therefore to a higher reactivity.

The energy gaps between HOMO and LUMO orbitals (Additional file 1: Figure S3) offer information about chemical reactivity of a molecule, a small gap suggesting an easy electronic transition and therefore a higher chemical reactivity. Chloronitro-compounds 1 and 2 have a larger energy gap than nitro-compound 3, which means that the electron transfer between the HOMO and LUMO orbitals is more possible to occur in the case of compound 3 than in the case of compounds 1 and 2.

In the same manner, by analyzing the variation of other descriptors of reactivity (Table 3) compound 3 seems to exhibit a higher reactivity since it has the highest electronegativity number (highest electron attraction tendency of molecules), the lowest chemical potential value (lowest escaping tendency of the electrons) and the highest electrophilicity index (highest electrophilic behavior) compared to its chlorinated derivatives.

**Table 3:** Chemical reactivity descriptors

Compound	Toxicity (logMRC50)	E_HOMO_ (eV)	E_LUMO_ (eV)	E_gap_ (eV)	µ (eV)	χ (eV)	ω (eV)
1	3.10	− 8.77077	2.20467	10.975	− 3.283	3.283	0.982
2	3.22	− 9.08751	1.61091	10.698	− 3.738	3.738	1.306
3	1.78	− 6.71305	− 4.72281	1.99	− 5.718	5.718	16.43

When the descriptors illustrating the chemical stability were analysed (Table 4), we noticed that the chloro-derivatives had a more negative value for the heat of formation and a lower dipole moment value, which indicates a higher stability compared with compound 3. Compound 3 also has a lower hardness value than the its chlorinated derivatives which makes it more susceptible to charge transfer and therefore more reactive. The chemical stability is usually associated to hard molecules.

**Table 4:** Chemical stability descriptors

Compound	Toxicity (logMRC50)	Heat of formation (kcal mol^−1^)	Dipole	Η (eV)
1	3.10	− 117.42	19.5258	5.488
2	3.22	− 121.19	17.5964	5.349
3	1.78	− 120.54	11.4651	0.995

**Fig. 5:**
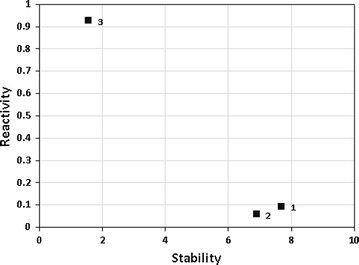
Distribution of compounds 1–3 based on their chemical reactivity and stability

We combined all the parameters describing the same characteristic of a molecule (reactivity or stability) in order to calculate a consensus score for each compound. We notice that compounds 1 and 2, which are more toxic, exhibit a higher stability and a lower reactivity (Fig. 5).

On the contrary, compound 3, which is less toxic, has a lower stability but a higher reactivity, although toxicity is usually associated with large values of reactivity [37]. In this light, probably the mechanism of action involves an accumulation of compounds 1 and 2 in the system since they do not react easily and are very stable.

### Anion stability and reactivity

A similar trend in chemical reactivity and stability variation was noticed if only the anions were considered (A1–A3), given that the same alkanolamine is present in all three compounds and the anion is the active component. Besides the quantum chemical descriptors which have already been discussed, the chemical reactivity and stability of an anion may be correlated with the its nucleophilic character and acidic strength. A measure of the nucleophilicity of a molecule is the ionization potential. A higher value of ionization energy, as in the case of A2 and A3, shows a higher attraction between the electron and the nucleus, therefore a lower reactivity (Table 5). The anion stability also depends on electron delocalization and aromaticity.

**Table 5:** Ionization potentials and acidic constants for anions A1–A3

Anion	Ionization potential (eV)	pKa^a^
A1	5.578062	1.92
A2	5.337785	2.17
A3	4.475456	3.43

^a^pKa of correspondent carboxylic acid [38]

The electron density of the anions is determined by the electronic effects of substitutents, so indirectly, if an acid is stronger, its conjugate base (anion) is weaker and more stable. Anion A3 is less stable, since the pKa of its conjugate acid is higher [38]. All three anions contain a nitro group with a resonance effect − R, that decreases the electron density at the aromatic ring, and thus it increases its inductive effect against carboxyl group, which can free easily the proton. 4-NO_2_BA and 2-Cl-4-NO_2_BA are stronger acids compared to 2-Cl-5-NO_2_BA due to the presence of nitro group in *para* position, which implies a greater closeness of the carboxyl group to the positive charge which occurs on the aromatic ring through conjugation with nitro group. If the positive charge is closer to the carboxyl, the electrons are shifted closer to the oxygen atom and the anion is more stable. Chlorine atom containing pairs of non-bonding electrons has a resonance effect + E against the aromatic ring, functioning as a strong electron donor. Implicitly, the − R effect of nitro group is mitigated by the presence of chlorine per core, so the anion of compound 3 is a stronger acid and therefore has greater stability than those with chlorine.

## Conclusions

Biological active salts with ethanolammonium as cation and 2-chloro-4-nitro/2-chloro-5-nitrobenzoate or 4-nitrobenzoate as anion were structurally, chemically and toxicologically investigated. Single crystal X-ray diffraction confirmed the proton transfer and ionic hydrogen bonds formation between the components. Therefore, in compounds 1 and 2, the components are linked only by one HB, while in compound 3 two charge-assisted HB are formed. Chloronitro compounds (1 and 2) adopt layered structure and nitrocompound (3) chain-like structure. The presence of chlorine atom in benzene rings has led to rotation of carboxyl groups with respect to this ring which forms the essentially non-planar anions of 1 and 2 in comparison with compound 3. The position of nitro-substituent leads to change in the system of hydrogen bonds between anion and cation in compound 2 which crystallizes as hydrate. The measured value of toxicity indicates a very low order of toxicity for compound 3 (1.78 log u.), almost half of the value of compound 1, respectively 2 (3.22 log u., 3.10 log u.) and lower compared to the individual components (4-NO_2_BA: 3.11 log u. and EA: 2.67 log u.). The theoretical study regarding chemical reactivity and stability profiles explains the experimental values of toxicity. Compound 3 showed a higher reactivity and a lower stability compared to its compound 1 and 2, which is in agreement with the lowest toxicity value measured in biological assay. In conclusion, toxicity test on *H. echinata* in relation with density functional theory, ab initio molecular orbital calculations and crystallographic study leads to a better understanding of nitro/chloronitro substituent effect on toxicity, contributing to the design of new compounds with low toxicity and practical applicability.

## Additional files

**Additional file 1.** Additional information includes UV-vis spectra, FT-IR spectra, selected bond lenghts (Å) and angles (°), the difference between HOMO and LUMO energies.

**Additional file 2.** Cif file for compound 1.

**Additional file 3.** Cif file for compound 2.
