# Supplementary material for: Synthesis, structure and toxicity evaluation of ethanolamine nitro/chloronitrobenzoates: a combined experimental and theoretical study
Source: Chem Cent J. 2017 Dec 6;11:129. doi: 10.1186/s13065-017-0346-5 (PMC5718998; doi:10.1186/s13065-017-0346-5)
Supplement: Supplementary file 1 — Additional file 1. Additional information includes UV-vis spectra, FT-IR spectra, selected bond lenghts (Å) and angles (°), the difference between HOMO and LUMO energies. [file 13065_2017_346_MOESM1_ESM.doc]

**Additional information for:**

**Synthesis, structure and toxicity evaluation of ethanolamine nitro/chloronitrobenzoates: a combined experimental and theoretical study**

Manuela Crisan^a^, Liliana Halip^a^, Paulina Bourosh^b^, Sergiu Adrian Chicu^c^, Yurii Chumakov^b*^

^a^Institute of Chemistry Timisoara of Romanian Academy, 24 Mihai Viteazul Boulevard, Timisoara, 300223, Romania; ^b^Institute of Applied Physics, Academy of Sciences of Moldova, Academiei Street 5, Chisinau, MD - 2028, Republic of Moldova; ^c^50859 Köln, Siegstr. 4, Germany

* Corresponding Author: Prof dr. **Yurii Chumakov**

Institute of Applied Physics, Academy of Sciences of Moldova

Laboratory of Physical Methods of Solid State Investigation „T. Malinowski”

Academiei Street 5, Chisinau, MD - 2028, Republic of Moldova

Email: chumakov@phys.asm.md

Phone: (373 22) 73 81 54, Fax: (373 22) 73 81 49

**Results and discussion**

***Synthesis and characterization***

compound 1

277.93, 1.67

243

280

320

360

400

0.0

0.5

1.0

1.5

2.0

2.5

3.0

3.5

4.0

4.5

5.0

nm

A

compound 2

279.81, 1.74

compound 3

Figure S1. UV-vis spectra of compounds 1 - 3 in 0.1 M NaOH solution

Figure S2. FT-IR spectra of compounds 1 - 3 (red) and corresponding acids (black).

Table S1. Selected bond lengths (Å ) and angles (°) for compounds 1, 2 and 3.

| **Compound** | **1** | **2** | **3*** |
| --- | --- | --- | --- |
| N(1)–C(1) | 1.482(3) | 1.480(3) | 1.484(3) |
| O(1)–C(2) | 1.418(2) | 1.417(2) | 1.422(2) |
| C(1)–C(2) | 1.497(3) | 1.503(3) | 1.508(3) |
| Cl(1)–C(4) | 1.734(2) | 1.738(2) | - |
| N(2)–O(4) | 1.213(3) | 1.228(3) | 1.233(3) |
| N(2)–O(5) | 1.220(3) | 1.217(3) | 1.217(3) |
| N(2)–C(6)/C(7) | 1.475(2) | 1.465(2) | 1.474(2) |
| O(2)–C(9) | 1.245(2) | 1.237(2) | 1.246(2) |
| O(3)–C(9) | 1.237(2) | 1.254(2) | 1.259(2) |
| C(3)–C(8) | 1.389(2) | 1.389(2) | 1.388(2) |
| C(3)–C(4) | 1.393(2) | 1.395(2) | 1.344(2) |
| C(3)–C(9) | 1.514(2) | 1.516(2) | 1.520(2) |
| C(4)–C(5) | 1.384(2) | 1.391(2) | 1.386(2) |
| C(5)–C(6) | 1.375(3) | 1.371(3) | 1.374(3) |
| C(6)–C(7) | 1.376(3) | 1.383(3) | 1.381(3) |
| C(7)–C(8) | 1.384(3) | 1.385(2) | 1.393(2) |
| N(1)–C(1)–C(2) | 110.0(2) | 110.2(2) | 110.8(2) |
| O(1)–C(2)–C(1) | 106.9(1) | 111.8(2) | 112.2(2) |
| O(4)–N(2)–O(5) | 123.6(2) | 123.8(2) | 123.7(2) |
| O(4)–N(2)–C(6)/C(7) | 118.2(2) | 117.6(2) | 118.1(2) |
| O(5)–N(2)–C(6)/C(7) | 118.3(2) | 118.5(2) | 118.2(2) |
| C(8)–C(3)– C(4) | 118.3(2) | 117.8(1) | 119.5(1) |
| C(8)–C(3)–C(9) | 120.0(1) | 118.6(1) | 118.7(1) |
| C(4)–C(3)–C(9) | 121.7(2) | 123.6(1) | 121.8(1) |
| C(5)–C(4)–C(3) | 121.6(2) | 122.1(2) | 120.6(2) |
| C(5)–C(4)–Cl(1) | 118.7(1) | 117.0(1) | - |
| C(3)–C(4)–Cl(1) | 119.7(1) | 120.9(1) | - |
| C(6)–C(5)–C(4) | 117.7(2) | 119.6(2) | 118.0(2) |
| C(5)–C(6)–C(7) | 123.2(2) | 118.6(2) | 122.9(2) |
| C(5)/C(6)–C(6)/C(7)–N(2) | 118.0(2) | 118.8(2) | 118.9(2) |
| C(7)/C(8)–C(6)/C(7)–N(2) | 118.8(2) | 118.9(2) | 118.2(2) |
| C(6)–C(7)–C(8) | 117.8(2) | 122.4(2) | 118.2(2) |
| C(7)–C(8)–C(3) | 121.5(2) | 119.4(2) | 120.5 (2) |
| O(2)–C(9)–O(3) | 127.4(2) | 124.6(2) | 124.6(2) |
| O(2)–C(9)–C(3) | 116.7(1) | 116.9(1) | 116.9(1) |
| O(3)–C(9)–C(3) | 115.9(1) | 118.5(1) | 118.5(1) |

*crystallographic data of compound 3 [21] is presented here for comparison with compounds 1 and 2

***Experimental determination and theoretical investigation of toxicity behaviour***


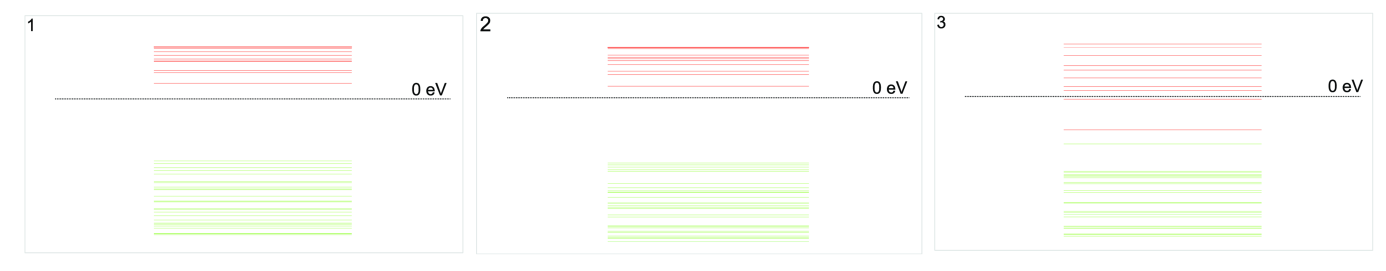


Figure S3. The difference between HOMO and LUMO energies for compounds 1-3
